# Laparoscopic versus Ultrasound-Guided Transversus Abdominis Plane Block for Postoperative Analgesia Management after Radical Prostatectomy: Results from a Single Center Study

**DOI:** 10.3390/jpm13121634

**Published:** 2023-11-23

**Authors:** Angelo Civitella, Francesco Prata, Rocco Papalia, Vincenzo Citriniti, Piergiorgio Tuzzolo, Giuseppe Pascarella, Ester Maria Alba Forastiere, Alberto Ragusa, Francesco Tedesco, Salvatore Mario Prata, Umberto Anceschi, Giuseppe Simone, Giovanni Muto, Roberto Mario Scarpa, Rita Cataldo

**Affiliations:** 1 Department of Urology, Fondazione Policlinico Universitario Campus Bio-Medico, 00128 Rome, Italy; a.civitella@policlinicocampus.it (A.C.); rocco.papalia@policlinicocampus.it (R.P.); p.tuzzolo@policlinicocampus.it (P.T.); alberto.ragusa@unicampus.it (A.R.); francesco.tedesco@unicampus.it (F.T.); r.scarpa@policlinicocampus.it (R.M.S.); 2 Department of Anesthesia and Intensive Care, Fondazione Policlinico Universitario Campus Bio-Medico, 00128 Rome, Italy; v.citriniti@policlinicocampus.it (V.C.); g.pascarella@policlinicocampus.it (G.P.); r.cataldo@policlinicocampus.it (R.C.); 3 Department of Anesthesia and Intensive Care, “Regina Elena” National Cancer Institute, 00144 Rome, Italy; ester.forastiere@ifo.it; 4 Simple Operating Unit of Lower Urinary Tract Surgery, SS. Trinità Hospital, Sora, 03039 Frosinone, Italy; mario.prata@libero.it; 5 Department of Urology, IRCCS “Regina Elena” National Cancer Institute, 00144 Rome, Italy; umberto.anceschi@gmail.com (U.A.); puldet@gmail.com (G.S.); 6 Department of Urology, GVM—Maria Pia Hospital, 10132 Turin, Italy; giov.muto@gmail.com

**Keywords:** minimally invasive, pain management, postoperative pain, radical prostatectomy, transversus abdominis plane block

## Abstract

(1) Background: Regional anesthesia, achieved through nerve blocks, has gained widespread acceptance as an effective pain management approach. This research aimed to evaluate the efficacy of laparoscopic (LAP) transversus abdominis plane (TAP) block in patients undergoing laparoscopic radical prostatectomy. (2) Methods: From January 2023 to July 2023, 60 consecutive patients undergoing minimally invasive radical prostatectomy were selected. Patients were split into two groups receiving ultrasound-guided (US) or laparoscopic-guided TAP block. The primary outcome was a pain score expressed by a 0−10 visual analog scale (VAS) during the first 72 h after surgery. (3) Results: Both LAP-TAP and US-TAP block groups were associated with lower pain scores postoperatively. No statistically significant differences were observed between the two groups in surgery time, blood loss, time to ambulation, length of stay, and pain after surgery (all *p* > 0.2). In the LAP-TAP block group, the overall operating room time was significantly shorter than in the US-TAP block group (140 vs. 152 min, *p* = 0.04). (4) Conclusions: The laparoscopic approach, compared to the US-TAP block, was equally safe and not inferior in reducing analgesic drug use postoperatively. Moreover, the intraoperative LAP-TAP block seems to be a time-sparing procedure that could be recommended when patient-controlled analgesia cannot be delivered.

## 1. Introduction

Since the advent of minimallyinvasive surgery (MIS) techniques such as laparoscopy and, more recently, robot-assisted surgery (RAS), there has been a significant surge in the adoption of these technologies in urology over the past two decades [1]. This trend encompasses a diverse array of urological procedures [2]. The implementation of laparoscopic and robotic surgery techniques has enhanced both patient-related outcomes and perioperative results across a range of complex urological surgical procedures. For this reason, urologists have been captivated by the idea of transitioning all their surgical procedures away from the ancillary open approach, like radical and partial nephrectomy, cystectomy with intracorporeal neobladder reconstruction, prostatectomy, pyeloplasty, and other reconstructive procedures [2]. The adoption of MIS, along with its acknowledged positive impact on functional and oncological outcomes, has brought about a revolution in the recommendations of international associations such as the European Association of Urology (EAU) and the American Urological Association (AUA), both of which now strongly advocate for MIS [3].

While laparoscopic and robotic surgeries typically lead to less pain, lower perioperative blood loss, and shorter hospital stays when compared to the open approach, patients may still encounter moderate pain at the incision sites [4]. Pain in the abdominal region following minimally invasive surgery may be attributed to various factors, including discomfort at the port-site incision, irritation of the diaphragm due to carbon dioxide (CO_2_) insufflation, and inadequate removal of insufflated gas. This postoperative pain can manifest in three distinct forms: incisional pain (pertaining to the surgical incision), visceral pain (deep-seated pain within the abdomen), and shoulder pain (a referred sensation originating from visceral pain). Postoperative discomfort constitutes a crucial element of perioperative care that could potentially lead to the onset of chronic pain. The use of analgesics may aid in expediting post-surgical recovery. Therefore, ensuring effective pain relief during the perioperative period is vital for enhancing a patient’s overall surgical experience.

Medical practitioners have been motivated to investigate alternative pain management approaches, moving beyond a sole reliance on opioids. This endeavor aims to reduce the potential complications that may arise from their usage. As part of multimodal pain management, the application of locoregional anesthesia is recommended on completion of classical analgesia techniques [5]. From this perspective, the employment of plane blocks for the abdominal wall has been widely demonstrated to reduce the frequency and intensity of postoperative pain following abdominal surgery.

Regional anesthesia, achieved through nerve blocks, has gained widespread acceptance as an effective pain management approach in various surgical disciplines. Reducing patient pain levels is a significant focus for healthcare providers, especially in light of regulatory constraints and the increasing emphasis on patient satisfaction in healthcare delivery. Specifically, transversus abdominis plane (TAP) blocks have demonstrated notable advantages in specialties like obstetrics/gynecology, bariatric, and colorectal surgery, among others. The somatic pain pathways, transmitted through the ipsilateral thoracolumbar fibers, are inhibited by TAP blocks. Firstly, described as a “blind” landmark-guided technique via the Petit triangle by Rafi A. N. [6], the target of the needle puncture is to inject the local anesthetic solution in the fascial plane between the internal oblique and transversus abdominis muscles, providing analgesia for the anterior-lateral abdominal wall [7]. Afterward, a TAP block has been described with various techniques designed to specifically target the thoraco-abdominal nerves, providing analgesia for the abdominal wall. In 2013, Favuzza et al. described a novel laparoscopic-guided approach to TAP blocks after the insertion of the optical trocar, which allows a direct vision of local anesthetic spread [8]. Furthermore, various research studies indicate that utilizing the TAP block not only relieves pain but also contributes to a decrease in opioid usage after surgeries such as general and gynecologic procedures, including hysterectomy, appendectomy, and cesarean section [9,10]. Although this technique has been demonstrated effective in reducing postoperative pain after abdominal surgeries [11,12,13], its implication in urologic surgery remains underdeveloped.

Radical prostatectomy actually represents the most performed procedure in urology through a minimally invasive approach as prostate cancer (PCa) is the most commonly solid organ malignancy diagnosed in med worldwide [14]. Against this background, we performed a comparative study between laparoscopic-guided (LAP) and ultrasound (US) guided TAP block for postoperative pain control. The primary outcome was the non-inferiority of the LAP-TAP in the postoperative pain control compared to the ultrasound TAP.

## 2. Materials and Methods

### 2.1. Study Population and Design

After internal ethical board committee approval, our institutional radical prostatectomy board-approved dataset was queried for “TAP block”, “prostatectomy”, and “laparoscopy”. From January 2023 to July 2023, all patients with a diagnosis of clinically localized PCa who elected to undergo laparoscopic radical prostatectomy (LARP) through a transperitoneal route with or without pelvic lymphadenectomy at our Institution were selected, enrolled in the study, and retrospectively analyzed. Patients were excluded in case of emergency surgery, American Society of Anesthesiologists Status Classification (ASA) score IV, history of allergy to local anesthetics, personal history of abdominal soreness due to the coexistence of other painful pathological conditions, or if they declined to consent to the procedure scheduled in the study. A proficient anesthesiologist with more than 10 years of experience in abdominal wall block, assisted by a senior resident, performed the same general anesthesia protocol on all patients. The same team performed the US-TAP block after induction of general anesthesia, just before patient draping. On the other hand, LAP-TAP blocks were performed by a single surgeon after a learning curve consisting of an initial series of 20 LAP-TAP blocks. Every patient was adequately informed of the procedural sequence of anesthesia and surgery and signed a dedicated informed consent before undergoing study procedures. Preoperative data included demographics, body mass index (BMI), ASA score, prostate-specific antigen (PSA) (ng/mL), ISUP Grade Group at the time of the diagnosis, previous abdominal surgeries, hypertension, diabetes, and El-Ganzouri score. Intraoperative data, including operative time, operating room occupancy time, estimated blood loss, postoperative opioid use, and time in the Post Anesthesia Care Unit (PACU), were collected. Patients were consecutively enrolled and assigned either to the US- or LAP-TAP block group following a 1:1 assignment scheme. The primary outcome of the study was the assessment of pain scores during the first 72 h after surgery. Postoperative pain score was expressed by a 0−10 visual analog scale (VAS) and measured in the first 72 h after surgery. Secondary outcomes included postoperative opioid consumption, operating room time occupancy, complications, time to ambulation, and total hospital length of stay (LOS).

### 2.2. Anesthesia Protocol

All the patients received a preemptive analgesia regimen with dexamethasone 8 mg, paracetamol 1000 mg, and ketorolac 30 mg intravenously; postoperative nausea and vomiting prophylaxis (PONV) included granisetron 3 mg and dehydrobenzoperidol 1.25 i.v. preoperatively. General anesthesia with tracheal intubation was induced using a standard pharmacologic regimen with Fentanyl 3–5 mcg/kg, Propofol 2.5 mg/kg, and Rocuronium 0.6 mg/kg. Anesthesia was maintained using sevoflurane 2% in a 50:50 oxygen:air mixture in order to obtain a minimum alveolar concentration (MAC) of 1. Continuous infusion of remifentanyl was administered, ranging from 0.03 to 0.08 mcg/kg/h; moreover, the grade of curarization was assessed by neuromuscular monitoring during all general anesthesia phases.

### 2.3. Surgical Technique

After sterile draping, all patients were positioned supine in steep Trendelenburg. Minimally invasive radical prostatectomy was performed in all cases through a transperitoneal route. After the introduction of the first optical laparoscopic trocar through a 12 mm sub-umbilical incision with an open access technique and the induction of 12 mmHg pneumoperitoneum, four more laparoscopic trocars (two 12 mm and two 5 mm ports) were placed according to a standard configuration for pelvic surgery. Radical prostatectomy was performed in a standard fashion, and vesicourethral anastomosis was performed using a modified Van Velthoven technique as previously described [14]. The specimens were retrieved in an entrapment bag through the suprapubic incision of approximately 4 cm, previously used for one 12 mm port. Ultimately, the fascia was sutured for ports larger than 5 mm, and the skin was closed using subcuticular sutures.

### 2.4. Transversus Abdominis Plane Block Protocol and Technique

**Figure 1:**
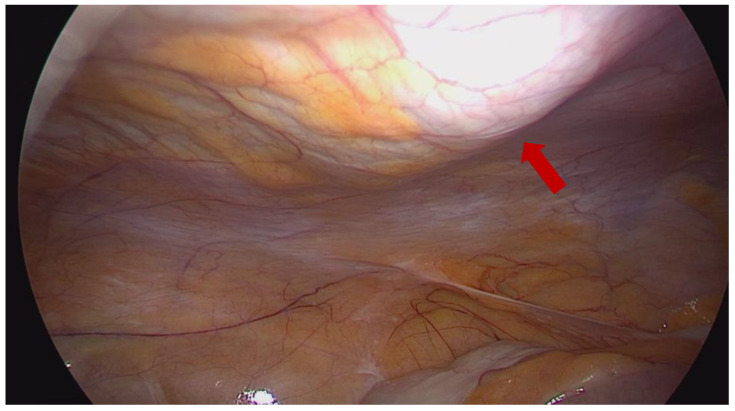
Direct visualization of the local anesthetic spread during minimally invasive radical prostatectomy. Red arrow showing the “virtual” location of the needle.

Both US-TAP and LAP-TAP blocks were performed at the anterior axillary line between the costal margin and iliac crest, injecting 20 mL of Ropivacaine 0.5% per side. In the LAP-TAP group, after the placement of the optical trocar, a 20G and 4′′ needle was inserted through the skin and progressed under laparoscopic vision until the needle pierced each of the 2 fascia layers (external oblique and internal oblique fascia). Thereby, the needle was positioned in the intramuscular plane between the internal oblique fascia and transversus abdominis muscle. At this point, 10 mL of local anesthetic was injected cranially towards the lower ribs, and 10 mL of the same mixture was injected toward the pelvic region using the same injection site. The injection was performed under continuous laparoscopic vision, assuring a smooth raised area of fluid covered by the transversus abdominis muscle without injection of the preperitoneal plane or peritoneal cavity (Figure 1).

In the US-TAP group, before surgery and right after sterile draping, a 22 G 80 mm echogenic needle (Stimuplex Ultra 360, BBraun, Melsungen, Germany) was inserted in a medial-to-lateral direction through an in-plane ultrasound approach and advanced towards the plane between internal oblique and transversus abdominis muscles. A high-frequency ultrasound transducer was placed in a transverse orientation at the midaxillary line, situated between the lower edge of the ribcage and iliac crest, to guide the positioning of the needle through the different abdominal layers. The correct placement of the needle tip was confirmed after aspiration by hydrodissection through a saline solution before the injection of local anesthetic. Both groups also received a wound infiltration with 10 mL of 0.5% ropivacaine. The total amount of ropivacaine for each group was 200 mg. LAP-TAP was performed by the same surgeon, while US-TAP was executed by the same group of anesthetists experienced in regional anesthesia. After extubation, every patient was transferred to the Post Anesthesia Care Unit PACU for postoperative monitoring. Pain intensity was assessed by a visual analog scale (VAS) from 1 to 10, and acute postoperative pain was treated with fentanyl boluses of 50 mcg. Discharge from PACU was admitted when adequate pain control was reached (mild pain, VAS < 4), absence of nausea and vomiting, hemodynamic stability, and adequate consciousness level.

### 2.5. Postoperative Management

In the postoperative phase, Acetaminophen 1000 mg was given to all patients on demand in case of VAS ≥ 4. Tramadol 100 mg was administered in case of moderate to severe pain, non-respondent to the aforementioned analgesics. Postoperative pain level was measured by our staff nurse using the visual analog scale (VAS), 0−10 scale, three times daily (6 a.m.–2 p.m.–10 p.m.) for the first 3 days during the hospital stay, along with routine vital signs assessment. All the nursing staff were blinded to the TAP block technique used for patients. Criteria for discharge were the absence of abdominal/pelvic pain, lack of medical and surgical complications, and normal kidney and bowel function (flatus and stool passing).

### 2.6. Statistical Analysis

The study population was split into two groups according to the TAP block technique used (US vs. LAP). Frequencies and proportions were used to report categorical variables that were compared by means of the Chi-squared test. Continuous variables were presented as median and interquartile ranges (IQRs) and were compared using either the Student’s *t*-test or Mann–Whitney U test based on their normal or not-normal distribution, respectively (normality of the distribution of variables was tested by the Kolmogorov-Smirnov test). A two-sided *p*-value < 0.05 was considered statistically significant. STATA (StataCorp. 2021. Stata Statistical Software: Release 17. College Station, TX, USA: StataCorp LLC.) was used for statistical analyses.

## 3. Results

Overall, 60 patients were enrolled (30 per each group). No statistically significant differences were recorded in terms of age, ASA, ISUP grade group, El-Ganzouri score, and BMI between the two groups (all *p* > 0.3). The descriptive characteristics of the two groups are listed in Table 1.

In the LAP-TAP and US-TAP groups, 18 and 17 patients underwent radical prostatectomy with contextual pelvic lymphadenectomy, respectively. An abdominal drainage was placed in all patients at the end of the procedure. The mean estimated blood loss was 148 mL in the LAP-TAP group and 145 mL in the US-TAP group, respectively. VAS assessments were performed to assess the quality of analgesia achieved in the study groups. There were no statistically significant differences in the VAS score between the two groups during the 72 h after surgery (Table 2, all *p* > 0.2).

In the recovery unit, the number of patients in each group who required at least a single administration of fentanyl analgesia was not significantly different, 6 for the US group and 7 for the LAP group (*p* = 0.75). No statistically significant differences were observed between the two groups in surgery time (100 min for the LAP-TAP block group and 102 min for the US-TAP block group, *p* = 0.66). In the LAP-TAP group, the overall operating room time was significantly shorter than in the US-TAP block group (140.9 vs. 153.1 min, *p* = 0.04). There were no significant differences in LOS, bowel recovery, use of opioids, and acetaminophen in the postoperative period (Table 2, all *p* > 0.5). No procedure-related adverse events, such as hematoma at the injection site, intravascular administration, or intraperitoneal injection, were recorded.

## 4. Discussion

Radical prostatectomy actually represents the most common surgical procedure performed in urology, either with an open approach or through laparoscopic- and robot-assisted techniques. Minimally invasive surgical procedures have become a cornerstone of perioperative care in surgical patients [15], as they deliver comparable oncological outcomes with potentially improved postoperative functional results, recovery, pain relief, and satisfaction. The continuous refinement of treatment options for the management of urological malignancies supports the patient-centered model of care based on the principle of informed and collaborative decision-making between patients and healthcare providers. Noteworthy, in this patient-tailored care system, comprehensive and multimodal management of the perioperative phase in patients undergoing minimally invasive radical prostatectomy still remains a poorly explored field.

Over the past ten years, there has been a growing focus on investigating how perioperative immunosuppression impacts the long-term oncological outcomes of patients. This is characterized by elevated glucocorticosteroid levels and compromised cellular and humoral immune responses. Numerous studies indicate that both postoperative pain and the use of opioids may contribute to perioperative immunosuppression [16,17]. However, recent evidence in the literature suggests that the adoption of Enhanced Recovery After Surgery (ERAS) protocols, along with strategies for pain management that reduce reliance on opioids, including regional anesthesia, could potentially alleviate this effect. According to ERAS protocol, in order to improve rehabilitation after major surgery, both surgical and anesthesiologic techniques should be as minimally invasive as possible [18].

Whenever it is feasible, minimally invasive procedures are recommended despite open technique, while perioperative pain management should be oriented to non-opioid analgesia [16,17,19]. MIS results in smaller surgical scars and potentially lowers postoperative pain, leading to a shorter recovery period. Anesthesiologists encounter novel challenges in patient care as well. Improved outcomes in MIS, especially concerning postoperative pain and recovery duration, are frequently attained through the integration of the minimally invasive approach with a customized multimodal anesthesia plan. Epidural anesthesia is widely recommended by ERAS protocols as a gold standard opioid-sparing technique to manage postoperative analgesia after major urologic surgery [20]. However, it is an invasive technique and may be related to different adverse events, such as postoperative headaches and hematomas, with consequent delay in hospital discharge [21,22]. In most recent years, the abdominal fascial plane block has been shown to have a comparable analgesic effect and less procedure-related risk of complications than the neuraxial technique [23]. The TAP block leads to a decrease in the use of postoperative narcotics and alleviates nausea and vomiting. It also enhances respiratory function, boosts patient contentment, and plays a role in enabling early patient mobilization and discharge.

The utilization of TAP block Is not as prevalent in urologic surgery, and there is a limited availability of studies that specifically examine minimally invasive radical prostatectomy within the context of enhanced recovery programs. While there may not be a substantial number of studies focusing on regional pain management in minimally invasive urologic surgery, it is clear that urologists are beginning to recognize the potential advantages that TAP blocks could offer, drawing inspiration from practices in other specialties. Nonetheless, growing evidence has emerged affirming its efficacy for various abdominal surgical procedures. Furthermore, the progression of US technology has made performing TAP blocks technically more feasible and safer. Two randomized controlled trials examined the role of TAP block in patients undergoing robot-assisted radical prostatectomy (RARP) [24,25] and concluded that TAP block was associated with a decrease in mean postoperative pain in the first 24 h, reduced opioid use, and a shorter length of stay. Comparable results on the efficacy of the TAP block were equally highlighted by two recent studies conducted by Rogers et al. and Chiancone et al. [26,27], in which the effectiveness of the US-TAP on postoperative pain and early walking resumption was evaluated. In both studies, a reduction in the use of painkillers in the postoperative period and an early resumption of walking compared to placebo was highlighted. Although US-guided TAP is the most commonly in the literature, recently, novel laparoscopic-guided techniques have been described and performed in different abdominal surgeries, including colorectal and gynecological surgeries, demonstrating a non-inferiority in terms of postoperative analgesia and opioid consumption [11,12,13]. Conversely, evidence about the use of TAP in urologic surgery is still missing. Ours is the first study aimed to compare the effectiveness of US and LAP-TAP under direct vision after the insertion of the optical trocar according to the technique described by Favuzza et al. [8]. Of note, no difference was reported between the two groups under examination in terms of operative time, postoperative pain, use of painkillers, LOS, and mobilization. Our results advocate the findings from previous studies comparing US-TAP with LAP-TAP in other abdominal laparoscopic surgeries [28]. Furthermore, our study shows a significant reduction in the operating room occupancy time in the LAP-TAP group with potential implications in time-saving procedures to enhance patient turnover. This could be related to the time needed for preparation and usage of the US-guided technique, considering that US machines may not be present simultaneously in all the operating theatres of the same hospital. This difference may reflect a cost saving for the hospital, where the operating room accounts for up to 40% of a hospital’s costs. With a cost conservatively estimated at USD 15–USD 50/minute [28], in this setting, a reduction in operating room time may potentially have an important impact on the hospital’s financial savings [29]. Moreover, the LAP-TAP block has multiple advantages: the video-laparoscopic visualization helps in directing the local anesthetic cranially and/or caudally toward the pelvis, ensuring a wider area of spread; it is an easy-to-learn technique, anatomical landmark visualization could improve the localization of the exact injection point, but the needle tip is not directly visible; the spread of the injected anesthetic is visualized as a spatial diffusion while the US-assisted technique allows a single slice visualization. Furthermore, the procedural time of LAP-TAP is significantly shorter than the US technique, and there is no need for an ultrasound machine in the operating theatre. Finally, in obese patients, the US visualization could be challenging due to excessive subcutaneous fat and increased depth, resulting in a difficult TAP block execution [30].

Some limitations of the study need to be mentioned. This study was conducted on a relatively small number of patients. As a limitation, the LAP-TAP block does not allow direct needle tip visualization, while the correct tip localization depends on the surgeon’s sensitivity to the “pop” which ensures the correct fascial plane localization, and this is indirectly confirmed by the visualization of the local anesthetic spread through video-laparoscopy. Moreover, surgeons and anesthesiologists were not blinded to the interventions, and postoperative pain was not assessed on movements. In addition, the retrospective nature of the study and the single-center experience could prevent drawing generalizable results.

Notwithstanding the acknowledged limitations, the results of this study confirm the non-inferiority of LAP-TAP block in comparison to the US-guided in terms of postoperative pain management. This study proved the ability to decrease the need for postoperative pain relievers, and this holds significant importance for several reasons. Firstly, the sometimes-spread misuse of opioids during the postoperative phase has emphasized the need to maximize pain relief while minimizing the reliance on medications, particularly narcotics. Secondly, even non-narcotic pain relievers come with known risks, such as acute kidney injury (e.g., dipyrone), liver failure (e.g., acetaminophen), and the potential for serotonin syndrome with the use of tramadol. Therefore, reducing the consumption of pain relievers can help mitigate the risks associated with these avoidable toxicities. Lastly, optimizing postoperative pain management is vital for implementing early discharge protocols, thus reducing hospital stays. This not only leads to lower complication rates but also results in potential healthcare cost savings.

Further prospective multicentric studies on a larger cohort are eagerly awaited to clearly define the role of the LAP-TAP block for urological pelvic procedures, such as for radical prostatectomy, and if this technique can improve and widen the application of the TAP block, particularly in obese patients, where the enhanced echogenicity of fatty tissue may prevent a satisfactory visualization of the fascial layer, avoiding a proper administration of analgesics.

## 5. Conclusions

In our experience, the LAP approach, compared to the US-TAP block in radical prostatectomy, seems to be equally safe and not inferior for postoperative pain control. Moreover, the LAP-TAP block may be a time-sparing procedure potentially cost-saving for the hospital, improving patient turnover and operating room occupancy. Notwithstanding our promising results, further prospective studies with a larger population are expected to externally validate the efficacy of LAP-guided TAP block in urologic pelvic surgery.

## Figures and Tables

**Table 1:** Descriptive characteristics of study populations.

	Entire Cohort	US-TAP Block	LAP-TAP Block	*p* Value
**Patient, n (%)**	60 (100)	30 (50)	30 (50)	
**Mean age, year (SD)**	67.9 (6.1)	67.8 (6.2)	68.1 (6.1)	0.77
**Mean BMI**	26.2	26	26.5	0.33
**PSA**	10.6 (5.7)	10.2 (5.6)	10.9 (5.8)	0.64
**EL-GANZOURI n°**				0.92
I	15	8	7	
II	25	11	14	
III	6	4	2	
IV	5	2	3	
V	7	4	3	
VI	2	1	1	
VII	0	0	0	
**ASA score n°**				0.69
I	5	4	2	
II	47	22	24	
III	8	4	4	
IV	0	0	0	
**ISUP Grade Groups n°**				0.97
I	13	6	7	
II	3	2	1	
III	16	8	7	
IV	25	12	13	
V	3	2	2	
VI	0	0	0	
**Previous abdominal surgery, n° (%)**	29 (48)	13 (43)	16 (53)	0.44

**Table 2:** Outcome comparison between ultrasound-guided and minimally invasive TAP block.

	US-TAP Block	LAP-TAP Block	*p* Value
Mean Operating room time, minutes, n° (SD)	153.1 (20)	140.9 (20.6)	**0.04**
Mean surgery time, n° (SD)	102.4 (19.6)	100.4 (19.8)	0.66
Mean length of surgical incision, cm (SD)	4.1 (0.5)	3.9 (0.5)	0.33
Mean PACU time (SD)	98.8 (26.7)	97.5 (27.5)	0.82
Fentanyl boluses (50 mcg) in the immediate postoperative time, n° (%)	6 (20)	7 (23)	0.75
Mean VAS Day 0, n° (SD)			
0 h	1.9	2.1	0.88
6 h	1.9	2.0	0.95
12 h	1.5	1.3	0.37
Mean VAS Day 1, n° (SD)			
06:00 a.m.	1.4	1.6	0.83
02:00 p.m.	0.9	0.9	0.96
10:00 p.m.	0.8	0.6	0.21
Mean VAS Day 2, n° (SD)			
06:00 a.m.	0.5	0.6	0.59
02:00 p.m.	0.5	0.3	0.20
10:00 p.m.	0.5	0.4	0.68
Mean VAS Day 3, n° (SD)			
06:00 a.m.	0.5	0.6	0.89
02:00 p.m.	0.5	0.5	0.96
10:00 p.m.	0.4	0.3	0.39
Acetaminophen 1 gr, n° (%)			
Day 0	6 (20)	8 (26)	0.54
Day 1	2 (7)	2 (7)	1
Day 2	1 (3)	0	0.31
Day 3	0	0	1
Tramadol 100 mg, n° (%)			
Day 0	0	0	1
Day 1	3 (10)	2 (7)	0.64
Day 2	0	0	1
Day 3	0	0	1
Prokinetics n° (%)			
Day 0	5	6	0.71
Day 1	6	5	0.71
Day 2	0	1	0.33
Day 3	0	0	1
Bowel recovery, days (SD)	1.5 (0.5)	1.6 (0.6)	0.87
Drain removal, days (SD)	2.2 (0.4)	2.3 (0.5)	0.77
LOS, days (SD)	4.1 (0.4)	4.2 (0.5)	0.56

## Data Availability

The data presented in this study is available on request from the corresponding author.
